# Diesterified Derivatives of 5-Iodo-2′-Deoxyuridine as Cerebral Tumor Tracers

**DOI:** 10.1371/journal.pone.0102397

**Published:** 2014-07-16

**Authors:** Thomas W. Rösler, Andreas Matusch, Damiano Librizzi, Oscar Arias-Carrión, Nils Freundlieb, Helmut Hoeffken, Wolfgang H. Oertel, Candan Depboylu, Günter U. Höglinger

**Affiliations:** 1 Experimental Neurology, Department of Neurology, Philipps-University, Marburg, Germany; 2 German Center for Neurodegenerative Diseases (DZNE), Munich, Germany; 3 Institute of Neuroscience and Medicine (INM-2), Research Center Jülich, Jülich, Germany; 4 Department of Nuclear Medicine, Philipps-University, Marburg, Germany; 5 Department of Neurology, Technical University, Munich, Germany; 6 Brain Imaging and Neurostimulation (BINS) Laboratory, Department of Neurology, University Medical Center Hamburg-Eppendorf, Hamburg, Germany; Biological Research Centre of the Hungarian Academy of Sciences, Hungary

## Abstract

With the aim to develop beneficial tracers for cerebral tumors, we tested two novel 5-iodo-2′-deoxyuridine (IUdR) derivatives, diesterified at the deoxyribose residue. The substances were designed to enhance the uptake into brain tumor tissue and to prolong the availability in the organism. We synthesized carrier added 5-[^125^I]iodo-3′,5′-di-*O*-acetyl-2′-deoxyuridine (Ac_2_[^125^I]IUdR), 5-[^125^I]iodo-3′,5′-di-*O*-pivaloyl-2′-deoxyuridine (Piv_2_[^125^I]IUdR) and their respective precursor molecules for the first time. HPLC was used for purification and to determine the specific activities. The iodonucleoside tracer were tested for their stability against human thymidine phosphorylase. DNA integration of each tracer was determined in 2 glioma cell lines (Gl261, CRL2397) and in PC12 cells *in vitro*. In mice, we measured the relative biodistribution and the tracer uptake in grafted brain tumors. Ac_2_[^125^I]IUdR, Piv_2_[^125^I]IUdR and [^125^I]IUdR (control) were prepared with labeling yields of 31–47% and radiochemical purities of >99% (HPLC). Both diesterified iodonucleoside tracers showed a nearly 100% resistance against degradation by thymidine phosphorylase. Ac_2_[^125^I]IUdR and Piv_2_[^125^I]IUdR were specifically integrated into the DNA of all tested tumor cell lines but to a less extend than the control [^125^I]IUdR. In mice, 24 h after i.p. injection, brain radioactivity uptakes were in the following order Piv_2_[^125^I]IUdR>Ac_2_[^125^I]IUdR>[^125^I]IUdR. For Ac_2_[^125^I]IUdR we detected lower amounts of radioactivities in the thyroid and stomach, suggesting a higher stability toward deiodination. In mice bearing unilateral graft-induced brain tumors, the uptake ratios of tumor-bearing to healthy hemisphere were 51, 68 and 6 for [^125^I]IUdR, Ac_2_[^125^I]IUdR and Piv_2_[^125^I]IUdR, respectively. Esterifications of both deoxyribosyl hydroxyl groups of the tumor tracer IUdR lead to advantageous properties regarding uptake into brain tumor tissue and metabolic stability.

## Introduction

The onward progress in the field of anti-tumor therapies led to increased interest in radiodiagnostics for selecting appropriate therapeutics and for the assessment of therapy success. [^18^F]fluorodeoxyglucose (FDG) in PET analysis is a gold standard for detecting vital tumor tissue, however, in the brain difficulties arise from the high physiologic uptake in healthy tissue resulting in decreased sensitivity and localizing validity for cerebral tumors.

Beside amino acid tracers [1]–[3], radiolabeled DNA building blocks, thymidine analogues in particular have been tested for localizing brain tumors with promising results [4], [5]. The fact that DNA synthesis is an essential and extensively accomplished process in cerebral tumors, renders labeled nucleoside analogues promising diagnostic tools for the visualization of proliferating tissue in imaging analysis. 3′-deoxy-3′-[^18^F]fluorothymidine ([^18^F]FLT) has been shown to be useful for differentiating low-grade from high-grade gliomas in PET analysis [4]. [^18^F]FLT is 5′-phosphorylated by thymidine kinase and accumulates intracellularly, but cannot be integrated into extending DNA because of the missing hydroxyl group in 3′-position. The thymidine analogue 5-iodo-2′-deoxyuridine (IUdR), in turn, takes fully part in DNA synthesis and is covalently incorporated in DNA [5].

IUdR was first synthesized by William Prusoff in 1959 [6] and tested as a radioiodinated tracer for DNA metabolism by Hughes et al. in 1964 who found IUdR to be a sensitive and specific tracer of DNA metabolism *in vivo* [7]. 5-[^124^I]iodo-2′-deoxyuridine ([^124^I]IUdR) was the first isotopologue tested in a human study [5]. The capacities of IUdR as mentioned above allow a narrow correlation of tracer uptake and DNA synthesis rate on the one hand and a good wash out of excessive unbound tracer resulting in a better signal-to-noise ratio on the other hand. Investigations on the metabolism revealed that IUdR tracers are rapidly degraded and that the elimination of the deoxyribose residue leading to 5-iodouracil (5-IU), and therewith to the loss of DNA integration properties, is the rate-limiting process [7]. To circumvent this obstacle a variety of IUdR analogues have been synthesized and tested *in vivo* for their abilities as tumor tracing agents. Most modifications to the IUdR structure were shown to be unsuitable because metabolism turned out to be even faster, or the DNA incorporation efficiency to be considerably poorer [8]. IUdR derivatives modified with halogenations or methylations on the deoxyribose residue did not accumulate in proliferating tissue any longer [8]. An exchange of the ring oxygen atom in the deoxyribose leading to 4′-thio-2′-deoxyuridine derivatives revealed more promising results, yielding a high metabolic stability and acceptable DNA incorporation rates. However, these derivatives showed a reduced phosphorylation by thymidine kinase 1 implicating an increased background radioactivity and, furthermore, revealed a decreased cell uptake in different tumor cell lines [8], [9]. In conclusion, previous studies clearly demonstrate strict limitations in varying the structure of IUdR to attain useful new tumor tracing agents.

In the present study we produced two novel 3′,5′-diesterified derivatives of IUdR with the aim to develop suitable tracers for imaging cerebral tumors. Due to the higher lipophilicity of these compounds and therewith an intended higher blood-brain barrier permeability, we aimed to reach an increased uptake in brain tissue. The radio nucleosides, 5-[^125^I]iodo-3′,5′-di-*O*-acetyl-2′-deoxyuridine (Ac_2_[^125^I]IUdR) and 5-[^125^I]iodo-3′,5′-di-*O*-pivaloyl-2′-deoxyuridine (Piv_2_[^125^I]IUdR) and their corresponding alkylstannylated precursor molecules 5-tributylstannyl-3′,5′-di-*O*-acteyl-2′-deoxyuridine (Ac_2_Bu_3_SnUdR) and 5-tributylstannyl-3′,5′-di-O-pivaloyl-2′-deoxyuridine (Piv_2_Bu_3_SnUdR) were synthesized and chemically characterized for the first time. To find out whether Ac_2_[^125^I]IUdR and Piv_2_[^125^I]IUdR can be used as imaging tracers, we first aimed at finding out if the novel derivatives are stable in relation to enzymatic degradation and if the tracers are integrated in the DNA of growing tumor cells. By *in vivo* experiments in mice we wanted to investigate the biodistribution properties of each tracer and to measure the specific uptake in brain tumor tissue. The overall aim was to find out if diesterification of IUdR leads to beneficial properties for tracing brain tumors.

## Materials and Methods

### Ethics Statement

All animal experiments were accomplished according to the EU Council Directive 2010/63/EU and to the institutional guidelines of Philipps-University Marburg and have been approved by the responsible authority (Regierungspräsidium Gieβen; permit number V54-19c 20 15(1) MR 20/15 Nr. 101/2011). Animals were kept at 23±1°C under standard 12 h light-dark cycle with free access to food and water. The animals were sacrificed by a single i.p. injection of 300 mg/kg pentobarbital. All surgery was performed under ketamine-xylazine anesthesia, and all reasonable measures were undertaken to prevent or to keep animal suffering to a minimum.

### General

Chemicals used for syntheses were obtained from Sigma Aldrich (St. Louis, MO) and Alfa Aesar (Ward Hill, MA) in chemical purities of ≥95%. Solvents applied in HPLC analyses were purchased from Merck (Darmstadt, Germany), Acros Organics (Geel, Belgium) and Sigma-Aldrich in gradient grade quality. ***HPLC:*** All HPLC analyses were conducted on a 600E multisolvent delivery system connected with a photodiode array detector 991 from Waters (Milford, MA). ***NMR:*** NMR spectra were recorded on a JEOL-ECA 500 (500 MHz) NMR spectrometer (Akishima, Tokyo, Japan). The values of the chemical shifts are given in parts per million (ppm) and are related to the δ- scale. The solvent signal was used as internal reference.

### Synthesis of 5-Iodo-3′,5′-di-*O*-acetyl-2′-deoxyuridine (Ac_2_IUdR)

5-iodo-2′-deoxyuridine (1.50 g; 4.24 mmol) was dissolved in 30 mL pyridine and acetic anhydride (3.90 mL; 41.25 mmol) was added slowly. The resulted mixture was stirred for 24 h at room temperature, subsequently poured in 150 mL of cold 2 N HCl and extracted with ethyl acetate. The organic layer was washed with saturated NaHCO_3_ and NaCl aqueous solutions and dried subsequently over anhydrous Na_2_SO_4_. After filtration and evaporation of the solvent, the product, a white solid, was recrystalized in ethanol yielding 1.66 g (90%). **^1^H NMR** (500 MHz, CDCl_3_) δ 2.01 (s, 3H, CH2″″); 2.08 (m, 1H, CH2′*); 2.10 (s, 3H, CH2‴); 2.43 (m, 1H, CH2′*); 4.19 (q, 1H, CH4′); 4.23 (dd, 1H, CH5′*); 4.30 (dd, 1H, CH5′*); 5.23 (m, 1H, CH3′); 6.28 (t, 1H, CH1′); 7.96 (s, 1H, CH4); 8.35 (bs, 1H, NH). **^13^C NMR** (500 MHz, CDCl_3_) δ 21.0 (C2‴); 21.3 (C2″″); 38.5 (C2′); 63.9 (C5′); 68.9 (C5); 74.2 (C3′); 82.9 (C1′); 85.6 (C4′); 143.9 (C6); 149.6 (C2); 159.4 (C4); 170.3 (C1‴); 170.5 (C1″″).

### Synthesis of 5-Iodo-3′,5′-di-*O*-pivaloyl-2′-deoxyuridine (Piv_2_IUdR)

5-iodo-2′-deoxyuridine (1.50 g; 4.24 mmol) was dissolved in 30 mL pyridine and trimethylacetyl chloride (1.305 mL; 10.59 mmol) was added slowly at 0°C. The mixture was stirred for 24 h at room temperature, subsequently poured in 120 mL ice water and extracted 3 times with equal volumes of chloroform. The unified organic layers were washed with 0.1 N HCl and the solvent was evaporated. The residue was purified by preparative HPLC (method A) to give 664 mg (30%) of a white solid as product. **^1^H NMR** (500 MHz, CDCl_3_) δ 1.22 (s, 9H, CH3‴); 1.26 (s, 9H, CH3″″); 2.09 (m, 1H, CH2′*); 2.57 (m, 1H, CH2′*); 4.25 (m, 1H, CH4′); 4.31 (dd, 1H, CH5′*); 4.53 (dd, 1H, CH5′*); 5.18 (m, 1H, CH3′); 6.21 (m, 1H, CH1′); 7.88 (s, 1H, CH4); 8.26 (bs,1H, NH). **^13^C NMR** (500 MHz, CDCl_3_) δ 27.1 (C2‴); 27.6 (C2″″); 38.5 (C2′); 38.8 (C3‴); 39.0 (C3″″); 64.0 (C5′); 68.9 (C5); 74.1 (C3′); 83.4 (C1′); 85.7 (C4′); 143.5 (C6); 149.5 (C2); 159.5 (C4); 178.1 (C1‴); 178.2 (C1″″).

### Synthesis of 5-Tributylstannyl-2′-deoxyuridine (Bu_3_SnUdR)

5-iodo-2′-deoxyuridine (0.50 g; 1.41 mmol) was dissolved in 20 mL 1,4-dioxane and dichlorbis(triphenylphosphine)palladium(II) (25 mg; 0.04 mmol) and hexa-n-butylditin (1.54 g; 3.55 mmol) were added. The mixture was heated at 120°C for 5 h under a stream of nitrogen. After cooling the mixture was filtrated and the solvent was removed by rotary evaporation. The resulted residue was separated by preparative HPLC (method A) to give 180.3 mg (25%) of the pure product; a thick, yellow opalescent oil. **^1^H NMR** (500 MHz, DMSO-d6) δ 0.82 (t, 9H, CH4″); 0.94 (m, 6H, CH1″); 1.24 (sex, 6H, CH3″); 1.44(m, 6H, CH2″); 2.06 (m, 2H, CH2′*); 3.50 (t, 2H, CH5′*); 3.76 (q, 1H, CH4′); 4.20 (m, 1H, CH3′); 4.88 (t, 1H, OH5′); 5.19 (d, 1H, OH3′); 6.15 (t, 1H, CH1′); 7.51 (t, 1H, CH4); 11.04 (s, 1H, NH); **^13^C NMR** (500 MHz, DMSO-d6) δ 9.9 (C1″); 14.1 (C4″); 27.1 (C3″); 29.0 (C2″); 40.4 (C2′); 62.1 (C5′); 71.3 (C3′); 84.7 (C1′); 88.0 (C4′); 111.5 (C5); 144.9 (C6); 151.4 (C2); 166.1 (C4).

### Synthesis of 5-Tributylstannyl-3′,5′-di-*O*-acteyl-2′-deoxyuridine (Ac_2_Bu_3_SnUdR)

5-iodo-3′,5′-di-*O*-acetyl-2′-deoxyuridine (1.00 g; 2.28 mmol) was dissolved in 40 mL 1,4-dioxane and hexa-*n*-butylditin (3.2 mL, 6.2 mmol) and dichlorbis(tri-phenylphosphine)-palladium(II) (40 mg; 0.06 mmol) were added. The mixture was heated at 120°C for 5 h under a stream of nitrogen. After cooling, the solvent was removed by rotary evaporation. The resulted residue was separated by preparative HPLC (method A) to give 451 mg (33%) of the product; a yellowish oil. **^1^H NMR** (500 MHz, CDCl_3_) δ 0.88 (t, 9H, CH4″); 0.95–1.11 (m, 6H, CH1″); 1.25–1.39 (m, 6H, CH3″); 1.41–1,55 (m, 6H, CH2″); 2.09 (s, 3H, CH2″″); 2.11 (s, 3H, CH2‴); 2.18 (m, 1H, CH2′*); 2.48 (m, 1H, CH2′*); 4.24 (m,1H,CH4′); 4.28 (dd, 1H, CH5′*); 4.34 (dd, 1H, CH5′*); 5.21 (m, 1H, CH3′); 6.24 (m, 1H, CH1′); 7.18 (t, 1H CH4); 8.16 (bs, 1H, NH). **^13^C NMR** (500 MHz, CDCl_3_) δ 10.0 (C1″); 13.8 (C4″); 20.9 (C2‴); 21.0 (C2″″); 27.3 (C3″); 29.0 (C2″); 37.6 (C2′); 63.9 (C5′); 74.3 (C3′); 82.2 (C1′); 85.5 (C4′); 113.5 (C5); 142.6 (C6); 150.7 (C2); 165.8 (C4); 170.3 (C1‴); 170.4 (C1″″).

### Synthesis of 5-Tributylstannyl-3′,5′-di-*O*-pivaloyl-2′-deoxyuridine (Piv_2_Bu_3_SnUdR)

5-iodo-3′,5′-di-*O*-pivaloyl-2′-deoxyuridine (0.60 g; 0.88 mmol) was dissolved in 20 mL 1,4-dioxane and hexa-*n*-butylditin (1.3 mL, 2.5 mmol) and dichlorbis-(triphenylphosphine)-palladium(II) (20 mg; 0.03 mmol) were added. The mixture was heated at 120°C for 5 h under a stream of nitrogen. After cooling, the solvent was removed by rotary evaporation. The resulted residue was separated by preparative HPLC (method A) to give 216 mg (36%) of the pure product; a slightly yellow, amorphous solid. **^1^H NMR** (500 MHz, CDCl_3_) δ 0.88 (t, 9H, CH4″); 0.96–1,11 (m, 6H, CH1″); 1.22 (s, 9H, CH2″″); 1.27–1.37 (m, 6H, CH3″); 1.41–1.55 (m, 6H, CH2″); 1.99 (s, 9H, CH3″″); 2.17 (m, 1H, CH2′*); 2.52 (m, 1H, CH2′*); 4.21 (m, 1H, CH4′); 4.24 (dd, 1H, CH5′*); 4.36 (dd, 1H, CH5′*); 5.18 (m, 1H, CH3′); 6.16 (m, 1H, CH1′); 7.21 (t, 1H, CH4); 8.17 (bs, 1H, NH). **^13^C NMR** (500 MHz, CDCl_3_) δ 10.0 (C1″); 13.8 (C4″); 27.1 (C2‴); 27.3 (C3″); 27.6 (C2″″); 29.0 (C2″); 37.8 (C2′); 38.7 (C3‴); 38.9 (C3″″); 63.9 (C5′); 74.1 (C3′); 82.5 (C1′); 85.7 (C4′); 113.1 (C5); 142.5 (C6); 150.6 (C2); 165.9 (C4); 178.0 (C1″″); 177.9 (C1‴).

### HPLC

#### Method A

The reaction product was dissolved in eluent (dichlormethane-isopropanol, 90∶10, v/v) at a maximum concentration of 200 mg/mL. 1 mL was subjected to a preparative column containing a SiOH normal phase (Nucleodur VarioPrep, 5 µm, ID 21 mm, L 250 mm Macherey-Nagel, Düren, Germany). An isocratic elution was carried out at a flow rate of 20 mL/min over 20 min.

#### Method B

The residue was dissolved in 100 µL eluent (acetonitrile-water, 90∶10, v/v) and 50 µL were subjected to an aminopropyl-modified normal phase (Nucleodur NH_2_, 5 µm, ID 4 mm, L 250 mm; Macherey-Nagel) carrying out an isocratic elution at a flow rate of 0.5 mL/min over 15–20 min.

#### Method C

The evaporated samples were dissolved in 40 µL eluent (acetonitrile-water, 80∶20, v/v) and 20 µl were subjected to an ammonium-sulfonic acid modified phase (Nucleodur Hilic, 5 µm, ID 4 mm, L 250 mm; Macherey-Nagel) with isocratic elution conditions at a flow rate of 1.2 mL/min over 5 min.

### Stability of the N-glycosidic Bond

The stability of the N-glycosidic bond was determined by *in vitro* incubation of the cold tracers with human recombinant thymidine phosphorylase (TP). Each iodonucleoside (IUdR, Ac_2_IUdR, Piv_2_IUdR) was dissolved in 150 mM K_2_HPO_4_ buffer solution (pH = 7.4) at a concentration of 0.67 nmol/µL. 150 µL of this solution were incubated with 0.5 µL TP solution (equal to 0.525 units, Sigma-Aldrich) for 0.5 and 6 h at room temperature (n = 4). After incubation, the reaction solution was heated at 90°C for 5 min, filtered and extracted 3 times with 200 µL ethyl acetate. The combined organic phases were evaporated and the residue was dissolved in eluent (acetonitrile/water; 80∶20) and submitted to HPLC analysis (Method C). The resulting metabolite 5-Iodouracil (5-IU) and intact tracers were quantitatively determined and correlated to untreated controls.

### Radiolabeling

The radiolabeling was carried out by using the method of Toyohara et al. 2002 [9] with modifications. In short, 0.7 mL water and 0.7 mL chloroform were poured into a reaction vial, 5 µL of a Na[^125^I]I (28 MBq in 0.1 N NaOH; Hartmann Analytic GmbH, Braunschweig, Germany) and 5 µL of a freshly prepared iodine solution (0.05 M, in chloroform) were added. After 10 s of vortexing, the aqueous phase was removed and 100 µL of the tracer precursor solution (Bu_3_SnUdR, Ac_2_Bu_3_SnUdR or Piv_2_Bu_3_SnUdR each dissolved in ethyl acetate at a concentration of 1.9 mM) were added. The mixture was vortexed for 10 s and allowed to react for 20 h. After evaporation of the solvent, the residue was dissolved in eluent and subjected to HPLC (method B). The amount of radiolabeled nucleoside was determined by peak area analysis referred to peaks of a serial dilution of cold tracer. The labeling yield was 47% for [^125^I]IUdR, 39% for Ac_2_[^125^I]IUdR and 31% for Piv2[^125^I]IUdR with chemical purities of >99%. Specific activities were adjusted to 50 MBq/µmol.

### Cell Lines

Murine glioma cells (GL261 [10], [11]; provided by Prof. A. Pagenstecher, Philipps-University, Marburg, Germany) and glioma cells of the rat (CRL2397; from American Type Culture Collection, Manassas, VA) were cultured in Dulbecco's Modified Eagle's Medium (DMEM; Sigma-Aldrich), supplemented with 10% (v/v) fetal calf serum (FCS; Sigma-Aldrich) and 1% (v/v) penicillin/streptomycin solution (PAA Laboratories Inc., Westborough, MA). Pheochromocytoma cells of the rat (PC12; provided by Prof. C. Möller, Philipps-University, Marburg, Germany) were cultured in DMEM, supplemented with 10% horse serum (Life Technologies, Carlsbad, CA), 5% FCS and 1% (v/v) penicillin/streptomycin solution.

For growth analysis, cells were seeded in 600 mm dishes (Cellstar, Greiner Bio-One GmbH, Frickenhausen, Germany) at 2×10^5^ cells per dish in appropriate culture medium in triplicates. The cells were trypsinized each day on 5 consecutive days and counted using a counting cell chamber (Neubauer, Brand, Wertheim, Germany). The doubling times (DTs) were determined to be 17 h for CRL2397, 21 h for GL261 cells and 51 h for PC12 cells, respectively.

### DNA Incorporation

The DNA incorporation of each tracer was determined by using a quantitative extraction technique to separate small molecules, RNA, DNA and proteins as described before [9], [12]. For detailed description see supporting information (protocol S1).

### Relative Biodistribution

Wildtype C57BL/6 mice (Charles River GmbH, Sulzfeld, Germany), 9–11 weeks of age, were used for the experiments. Mice (n = 6 per tracer) were injected intraperitoneally (i.p.) with 0.5 MBq of ^125^I-labeled tracer dissolved in 0.1 mL of 0.9% NaCl solution and sacrificed 24 h later. After extensive transcardial perfusion with ice-cold 0.9% NaCl solution, the organs were dissected, weighed and their radioactivities were measured by a gamma counter (Cobra II, Perkin-Elmer Packard, Waltham, MA).

### Tumor-specific Uptake *in vivo*

Host wildtype C57BL/6 mice, 12–14 weeks of age were anesthetized with ketamine-xylazine (87 and 13 mg/kg, respectively), and fixed in a stereotaxic frame (Kopf Instruments, Tujunga, CA). A burr hole was drilled in the skull and GL261 cells were aspirated using 27-gauge needle attached to a 10 µL Hamilton syringe mounted in a manually driven micro injector (Kd Scientific, Holliston, MA). Each animal received 6 µL of DMEM, containing a suspension of 25.000 cells/µL, injected at a flow rate of 0.5 µL/min into the striatum (from bregma: anterior 0.0 mm, lateral 3.0 mm, ventral 5.0 mm, incisor bar 0, according to Franklin and Paxinos [13]). After 4 weeks, mice were injected i.p. with 1 MBq of ^125^I-labeled tracer (n = 3 mice per tracer) and sacrificed 24 h later. Animals were transcardially perfused with ice-cold 0.9% NaCl solution and brains were dissected. From each brain hemisphere, tumor-bearing and tumor-free side, defined pieces of tissue (tumoral and non-tumoral) were cut-out, weighed and their radioactivities were measured by a gamma counter (Cobra II).

## Results

### Synthesis and Radiolabelling

**Figure 1:**
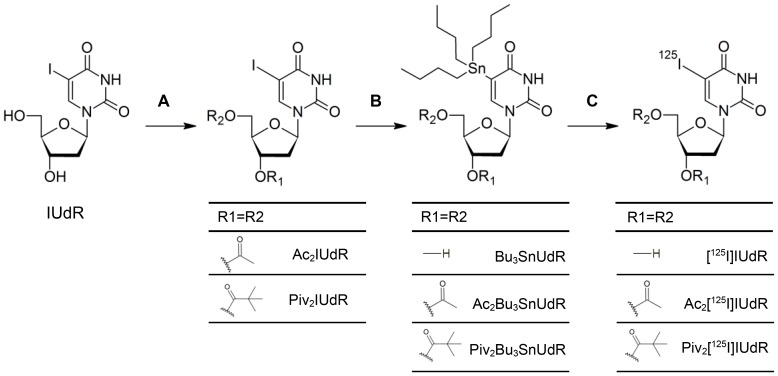
Synthetic pathway of the nucleoside tracers. (**A**) 5-Iodo-2′-deoxyuridine (IUdR) is esterified on both hydroxyl groups of the deoxyribose. (**B**) The diesterified derivatives are trialkystannylated at position 5 of the uracil ring. (**C**) The tributyltannyl group is selectively exchanged by iodine-125 (radioiodination).

Fig. 1 illustrates the synthesis routes of the radiolabeled nucleosides. Both hydroxyl groups of commercially available IUdR were esterified to give the diacetyl (Ac_2_IUdR) or dipivaloyl (Piv_2_IUdR) derivatives [14], [15]. In a so-called Stille reaction the substances were subsequently alkylstannylated with hexa-*n*-butylditin in the presence of a palladium (II) catalyst to give the corresponding tracer precursors (Bu_3_SnUdR, Ac_2_Bu_3_SnUdR and Piv_2_Bu_3_SnUdR). Due to their high lipophilicity and therewith sparse solubility in aqueous systems, iodination with conventional labeling methods e.g. chloramine T or H_2_O_2_-HCl were not useful or yielded too less radiolabeled product. Hence, a modified method of Toyohara et al. was used [9], wherein Na^125^I was first activated with iodine in a biphasic system of chloroform and water. After removal of the aqueous phase, the lipophilic iodonucleoside precursors, dissolved in ethyl acetate, could easily be added and reacted to the radiolabeled tracers (Ac_2_[^125^I]IUdR, Piv_2_[^125^I]IUdR and [^125^I]IUdR) with high specific activities ranging between 57 and 125 MBq/µmol.

### HPLC Separation

**Figure 2:**
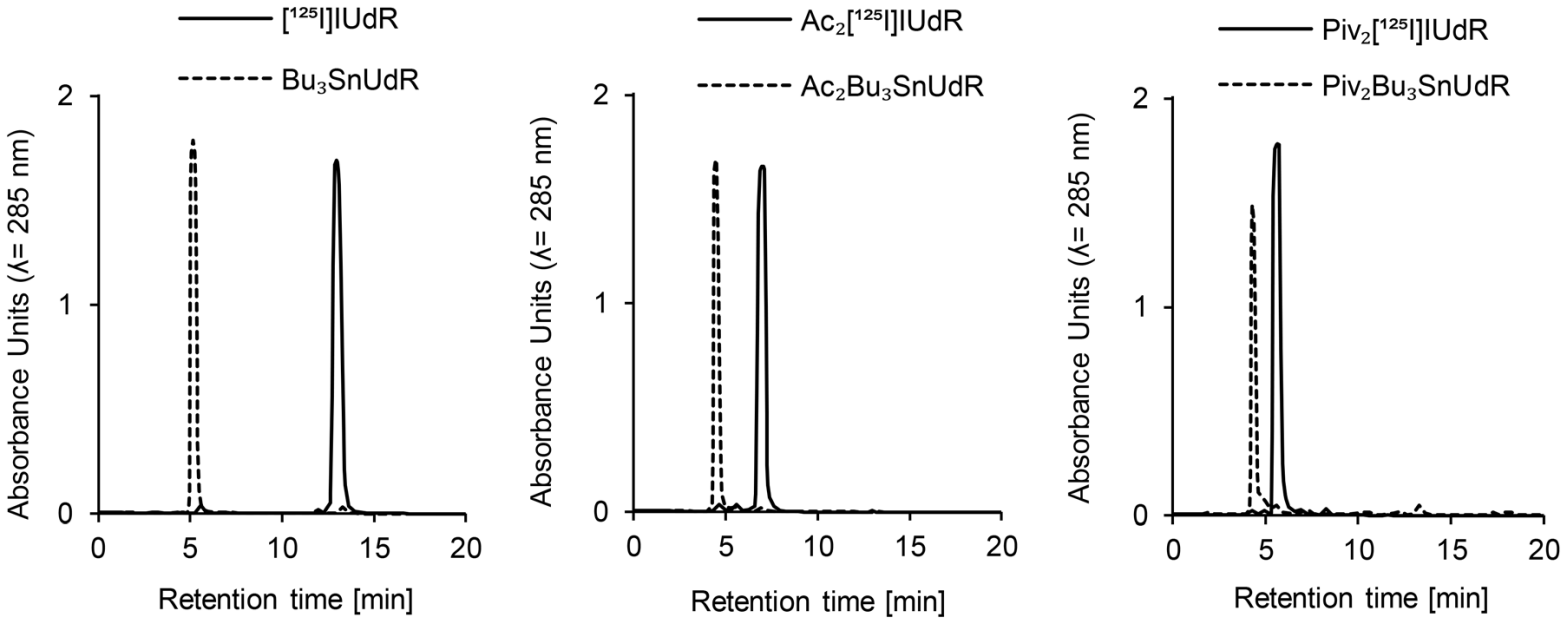
HPLC separation on an aminopropyl-modified normal phase. Merged HPLC chromatograms of each precursor and its corresponding iodine-125 labeled tracer (HPLC method B).

In order to separate the radiolabeled tracers from the unreacted precursor molecules and therewith to receive the tracers chemically pure, we tested different HPLC columns and conditions. We established a fast and straightforward HPLC method using a NH_2_ normal phase, facilitating to conduct the separation with a relatively low concentration of water and without addition of acid to the eluent (method B). For the sensitive esterified tracers, this approach guaranteed mild and gentle conditions during the HPLC process. By quantification of the peak areas and correlating them with standard concentrations, the amounts and the specific activities of produced radiolabeled tracers could be determined very precisely (Fig. 2).

### Susceptibility to N-glycosidic Bond Cleavage

Nucleoside tracers such as IUdR are highly susceptible to N-glycosidic bond cleavage by the enzyme thymidine phosphorylase. The cleavage leads to the metabolite 5-iodouracil and therewith to the loss of the DNA integrating abilities of the tumor tracer. In view of this, we investigated the stability of the synthesized tracers against thymidine phosphorylase *in vitro* (Table 1). In short (0.5 h) and long term (6 h) incubation conditions, the novel diesterified tracers were nearly resistant to N-glycosidic bond cleavage. Almost 100% of both diesterified tracer was still intact after 6 h of incubation, whereas the control IUdR was degraded completely.

**Table 1:** Susceptibility to N-glycosidic bond cleavage by thymidine phosphorylase after 0.5 and 6 h (n = 4) of incubation.

	0.5 h		6 h	
Tested nucleoside	Formed 5-Iodo-uracil [%]	Intact nucleoside [%]	Formed 5-Iodo uracil [%]	Intact nucleoside [%]
IUdR	100.95±3.92	<0.05*	99.28±1.42	<0.05*
Ac_2_IUdR	0.28±0.00	99.32±4.49	0.50±0.12	98.72±1.88
Piv_2_IUdR	0.07±0.00	100.18±2.05	0.23±0.08	99.90±3.50

values are given as percentage related to the amount of incubated iodonucleoside (mean ± SEM).

*experimentally determined detection minimum of the HPLC system.

### DNA Incorporation

**Figure 3:**
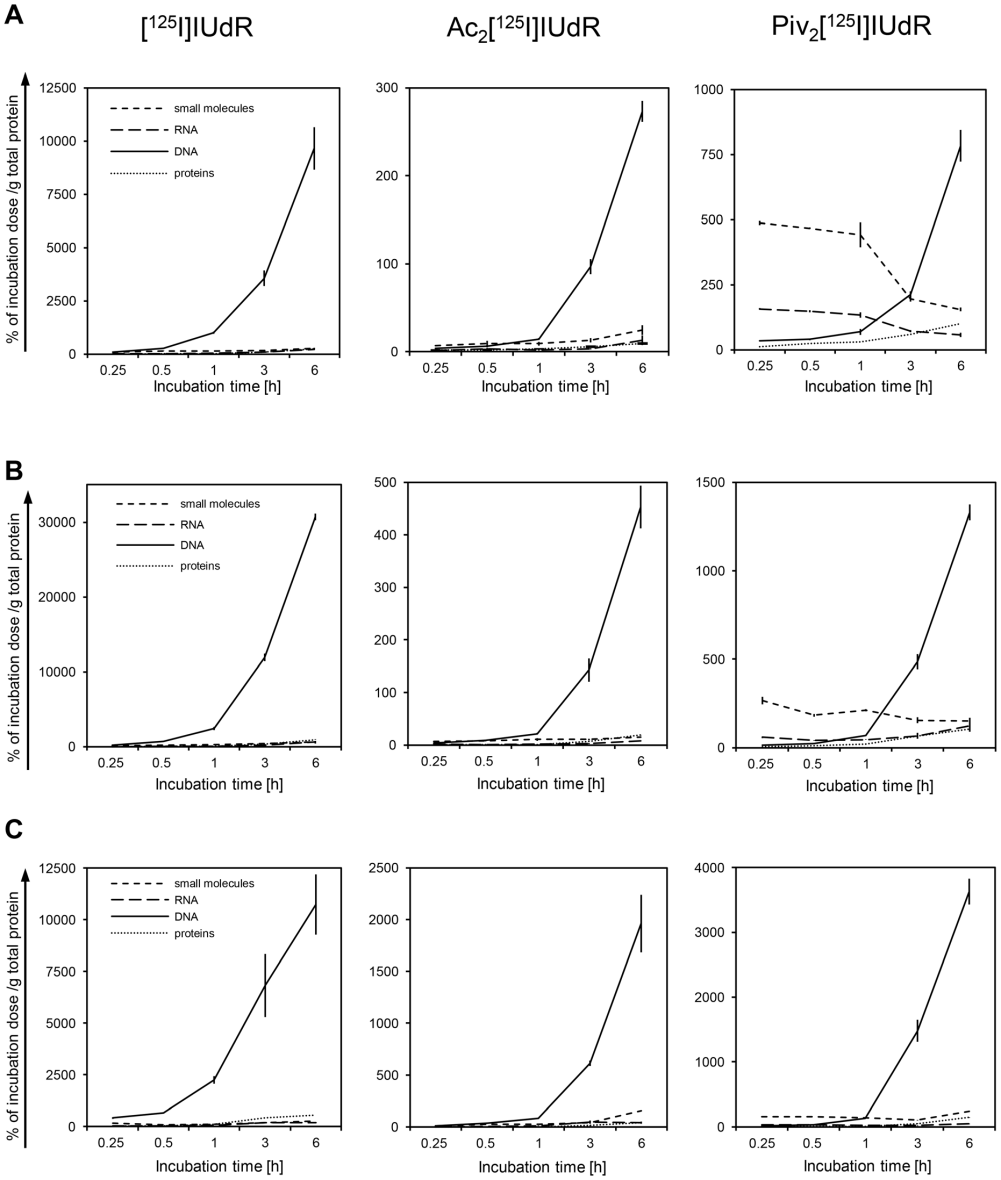
Time-dependent *in vitro* DNA incorporation of Ac_2_[^125^I]IUdR and Piv_2_[^125^I]IUdR compared to [^125^I]IUdR (control). The amount of each tracer was determined in the RNA, DNA, small molecule and protein fraction after incubation (for 0.25, 0.5, 1, 3 and 6 h) in 3 different tumor cell lines; (**A**) in glioma cells of the rat (CRL2397), (**B**) murine glioma cells (GL261) and (**C**) rat pheochromocytoma cells (PC12). The percentage of incubation dose was related to total protein. Values are presented as mean ± SEM (n = 3).

To determine whether the synthesized tracers are integrated in DNA, we performed *in vitro* incubation experiments with brain tumor and pheochromocytoma cells. Fig. 3 shows the time-dependent DNA incorporation of the synthesized tracers Ac_2_[^125^I]IUdR and Piv_2_[^125^I]IUdR compared to the control [^125^I]IUdR in fast-growing glioma cells of the rat (CRL2397; DT 17 h) and mouse (GL261; DT 21 h), and in moderate-growing pheochromocytoma cells of the rat (PC12; DT 51 h). The DNA incorporation of each tracer followed an almost exponential like curve when plotted over incubation time. Both synthesized tracers were well integrated in DNA but did not reach the extent of the control tracer [^125^I]IUdR within the 6 h observation period. In comparison to [^125^I]IUdR, the DNA incorporation curves of either diesterified tracer steepened (passed approx. 10% of the 6 h uptake value) after a lag phase (beyond 1 h) probably resulting from the ester cleavage activation step the tracers had to undergo before being integrated in DNA. In the RNA, small molecules and protein fractions, the tracers were only measurable on a baseline level, except for Piv_2_[^125^I]IUdR which had entered the small molecule fraction of CRL2397 cells already at the first time point (15 min) but persisted there to declining degree.

### Relative Organ Distribution

To investigate the distribution behavior and the stability *in vivo*, we applied the ^125^I-labeled tracers to healthy mice and measured the radioactivities in different body organs after 24 h of exposure (Table 2). Overall, Ac_2_[^125^I]IUdR and Piv_2_[^125^I]IUdR were similarly distributed as the control [^125^I]IUdR, but especially Ac_2_[^125^I]IUdR showed some interesting differences. In Ac_2_[^125^I]IUdR injected animals we detected decreased amounts of radioactivities in critical organs such as spleen and femur (hematopoietic tissue), stomach, intestine and lung. This uptake profile promises advantageous dosimetry in humans. In addition, the lower uptake of Ac_2_[^125^I]IUdR in the thyroid can be due to an enhanced *in vivo* stability towards deiodination. In comparison to [^125^I]IUdR, both diesterified tracers showed a slightly higher accumulation in the brain correlating with the grade of lipophilicity.

**Table 2:** Relative radioactivity distribution of iodonucleosides 24/6 Mice (n = 6 for each tracer).

Organ	[^125^I]IUdR	Ac_2_[^125^I]IUdR	Piv_2_[^125^I]IUdR
Blood	3.96±1.06	4.15±2.77	3.90±0.93
Kidney	1.71±0.37	1.71±0.59	1.84±0.43
Spleen	4.60±0.54	3.48±0.64	2.81±0.59
Femur	2.98±0.49	2.06±0.38	2.49±0.64
Thyroid	17.24±4.23	7.49±3.09	21.87±7.69
Intestine	10.27±1.13	6.64±0.95	10.77±2.52
Stomach	30.10±8.58	14.21±4.27	37.15±10.09
Liver	1.17±0.15	1.12±0.38	1.21±0.30
Heart	0.46±0.07	0.41±0.17	0.50±0.13
Brain	0.05±0.01	0.06±0.03	0.08±0.02
Muscle	0.54±0.12	0.77±0.52	0.56±0.11
Lung	1.44±0.45	0.86±0.17	2.17±1.02

values are given in percentage of injected dose per gram ×10^−1^ (mean ± SEM).

### Uptake into Cerebral Tumors

Next, we wanted to evaluate the specific uptake of the diesterified tracers in tumor tissue *in vivo*. Therefore we implanted murine glioma cells (GL261) into one brain hemisphere of mice. After 4 weeks of tumor growth, we measured the uptake of each tumor tracer in the tissue of the tumor-bearing compared to tumor-free hemisphere (Table 3). Ac_2_[^125^I]IUdR and Piv_2_[^125^I]IUdR as well as the control tracer [^125^I]IUdR showed a specific affinity for the tumor-bearing side. Ac_2_[^125^I]IUdR revealed the highest amount of tracer in the tumor-bearing hemisphere. By comparing the ratios of tumor-bearing to tumor-free side, the amount of Ac_2_[^125^I]IUdR was nearly 68 times higher in the tumor-bearing side, whereas for the control tracer [^125^I]IUdR this ratio was only 51. This result may be an indication of a higher specific uptake of the Ac_2_[^125^I]IUdR into cerebral tumor tissue.

**Table 3:** Uptake of iodonucleosides in tumor tissue *in vivo* (n = 3 for each tracer).

Iodonucleoside	Tumor-bearing hemisphere*	Tumor-free hemisphere*	Ratio tumor-bearing/tumor-free hemisphere
[^125^I]IUdR	4.45±1.41	0.09±0. 05	51
Ac_2_[^125^I]IUdR	11.16±4.36	0.17±0.04	68
Piv_2_[^125^I]IUdR	1.07±0.27	0.18±0.05	6

*values are given in percentage of injected dose per gram ×10^−3^ (mean ± SEM).

## Discussion

Previous studies identified three important aspects that need to be considered for the development of novel cerebral tumor tracers based on the IUdR structure. First, the two hydroxyl groups of the deoxyribosyl group in the IUdR structure are obligatory to ensure the integration in DNA, second, an iodination of the uracil ring at positions other than position 5 causes instability and third, modifications at the deoxyribosyl group e.g. halogenation decrease the efficiency of DNA integration [8], [9], [16], [17]. These limitations only allow minor structural modifications.

In the present work, we synthesized IUdR tracers with two esterified hydroxyl groups of the deoxyribose ring. Therefore, we first produced the diacetyl (Ac_2_IUdR) and dipivaloyl (Piv_2_IUdR) derivatives which were then trialkylstannylated in position 5 of the uracil ring to give the tracer precursors Ac_2_Bu_3_SnUdR and Piv_2_Bu_3_SnUdR. The alkylstannylation was necessary to guarantee the radiolabeling with iodine at this position. Additionally, it offers the chance to produce the radiolabeled tracers in a one-pot synthesis without further purification for *in vivo* application, since the tributylstannyl group, which is cleaved during the radiosynthesis, has a relatively low toxicity [18].

The tracer precursors were radiolabeled with iodine-125 which was produced by Na^125^I and iodine in a biphasic system of chloroform and water. This method, which has been described previously [9], was best suited for labeling the lipophilic precursors. By changing certain parameters, e.g. decreasing the amount of water and chloroform or prolonging the reaction time, we received higher labeling yields. Due to the longer reaction time it was not necessary to stop the reaction by adding sodium metabisulfite solution, thus the tracers were not additionally adulterated with salts.

Metabolic stability is an important issue in the development of new tumor tracers. For tracers based on IUdR the presence of the enzyme thymidine phosphorylase (TP) is pivotal because it rapidly cleaves the N-glycosidic bond which leads to tracer inactivation [19]. This circumstance gets worsened by the fact that TP is not only a ubiquitous enzyme but also occurs on upregulated levels in solid tumors and is associated with tumor aggressiveness [20]. By incubation experiments with TP under short and long term conditions we found that both diesterified tracers revealed a high stability of the N-glycosidic bond as compared to the control IUdR. Nearly 100% of intact tracer was still measurable after 6 h whereas IUdR was completely degraded within this time. In direct comparison of the diesterified tracers, the dipivaloyl ester (Piv_2_IUdR) showed a slightly higher resistance to TP than Ac_2_IUdR. From this finding can be concluded that big space-consuming ester groups on the deoxyribose residue sterically shield the C-N bond against enzymatic cleavage, and this effect seems to correlate with the size of the ester group.

Both tracers, Ac_2_[^125^I]IUdR and Piv_2_[^125^I]IUdR were specifically integrated into the DNA of 2 glioma cell lines and PC12 cells *in vitro*. Over time, DNA integration followed an almost exponential like pattern whereas the levels of integrated Ac_2_[^125^I]IUdR and Piv_2_[^125^I]IUdR were lower compared to the control tracer [^125^I]IUdR. An explanation for this result might be the activation step which the diesterified tracers must undergo before they can be integrated in DNA, namely the enzymatic hydrolysis of the esters. The activation step may also be the reason for a higher lag time of the DNA integration curves of the esterified tracers as compared to the control tracer [^125^I]IUdR.

By investigating the relative biodistribution of Ac_2_[^125^I]IUdR or Piv_2_[^125^I]IUdR in mice, we found a mostly similar distribution pattern as for the control [^125^I]IUdR. Interestingly, for Ac_2_[^125^I]IUdR, but not for Piv_2_[^125^I]IUdR, we measured intensively decreased amounts of tracer in the stomach and thyroid as compared to the control [^125^I]IUdR. This result implies a higher *in vivo* stability. Furthermore, the organ distribution of Ac_2_[^125^I]IUdR pointed to a lower whole body dose exposition. The amount of Ac_2_[^125^I]IUdR and Piv_2_[^125^I]IUdR were increased within the brains compared to animals which received [^125^I]IUdR. Although this result was not statistically significant, nevertheless, it suggests that lipophilic diesterified IUdR tracers enter the brain to a greater extent than the hydrophilic IUdR. Interestingly, lipophilic 5′-monoesterified IUdR derivatives have already been shown to be increasingly transported through the blood-brain-barrier in a previous study [21].

Both diesterified tracers, Ac_2_[^125^I]IUdR and Piv_2_[^125^I]IUdR revealed a specific uptake into the tumor-bearing brain side of mice which have been grafted with glioma cells. Ac_2_[^125^I]IUdR showed the quantitatively highest accumulation in the tumor-bearing side and showed a higher tumor-bearing to tumor-free ratio as compared to the control tracer [^125^I]IUdR. This ratio was much smaller for the more lipophilic Piv_2_[^125^I]IUdR which additionally showed a higher accumulation in the tumor-free side as compared to the other tracers.

Taken together, in the present study we synthesized two novel diesterified IUdR derivatives and tested their properties as tumor tracers in *in vitro* and *in vivo* experiments. Our results show that diesterification highly protects IUdR-tracers from metabolic degradation. Furthermore, the chemical modification led to higher amounts of diesterified tracers in healthy mice brains. We saw a specific accumulation of one diesterified tracer (Ac_2_IUdR) in the tumor bearing side of mice grafted unilaterally with brain tumor cells, whereas the specificity seems not only to be related to the fact of diesterification but also to the type of ester residue. The diesterified IUdR tracers were specifically integrated in DNA *in vitro*, although to a lower level than the control IUdR. The latter result raises the questions of how good the diesterified IUdR tracers are integrated in DNA *in vivo* and how suitable they are for labeling brain tumors in imaging analysis. These issues need to be addressed in further investigations.

## Supporting Information

Figure S1 **Atom numbering of chemical structures.** Overview of the atom numbering in the structure of the synthesized compounds for structural elucidation by NMR analysis.
(TIF)

Protocol S1 **DNA incorporation** ***in vivo*** **.**
(DOCX)
