# Supplementary material for: Diesterified Derivatives of 5-Iodo-2′-Deoxyuridine as Cerebral Tumor Tracers
Source: PLoS One. 2014 Jul 16;9(7):e102397. doi: 10.1371/journal.pone.0102397 (PMC4100890; doi:10.1371/journal.pone.0102397)
Supplement: Protocol S1 — DNA incorporation in vivo . (DOCX) [file pone.0102397.s002.docx]

**Protocol S1. DNA Incorporation *in vivo*.**

In short, cells (GL261, PC12 and CRL2397) were seeded at a concentration of 2.5 x 10^6^ in a 600 mm dish (Cellstar) for 24 h. After removal of the medium, 3 mL medium containing 0.4 MBq of idodine-125 labeled tracer were added to each dish and incubated at 37°C for 0.25, 0.5, 1, 3 and 6 h. Thereafter, the medium was removed, cells were washed twice with 2 mL ice-cold PBS, harvested by scraping and collected by centrifugation. 0.6 mL of ice-cold 0.2 N HClO_4_ was added and the cells were physically lyzed by 3 freeze-thaw cycles. The suspension was cooled on ice and centrifuged at 4°C and 1,200 *g* for 10 min. The acid-soluble fraction (small molecules) was removed, the precipitate resuspended in 1.2 mL of a 0.1 M HEPES (pH= 7.6) buffer solution with 0.66 mg/mL ribonuclease A (type III from bovine pancreas, 85-140 Kunitz units/mg; Sigma-Aldrich) and allowed to incubate at 37 °C for 30 min. Afterwards 0.3 mL of ice-cold 5 N HClO4 was added, the mixture was cooled and centrifuged. The supernatant (RNA-fraction) was separated and the precipitate was resuspended in 0.6 mL 1 N HClO4 and incubated at 90°C for 15 min. After cooling and centrifugation, the supernatant (DNA-fraction) was removed. The remaining pellet was washed with 1 mL ice-cold 1 N HClO4 and then solubilized in 0.4 mL 1 N NaOH to give the macromolecule fraction (proteins and lipids). The radioactivity of each fraction was then determined by a gamma counter (Cobra II, Perkin-Elmer Packard, Waltham, MA). The amount of proteins in the neutralized macromolecular fraction was quantified for standardization. Therefore 10 µL samples of each fraction were measured in a microplate assay by the Pierce Coomassie Plus Bradford Protein Assay Kit (Thermo Scientific, Waltham, MA). BSA (2 mg/mL) in a serial dilution (1:2) of 8 different concentrations was used as reference. The 96 well plates were readout by a microplate reader (Dynex MRX, Chantilly, VA).
